# We Know Too Little about Parasitoid Wasp Distributions to Draw Any Conclusions about Latitudinal Trends in Species Richness, Body Size and Biology

**DOI:** 10.1371/journal.pone.0032101

**Published:** 2012-02-15

**Authors:** Donald L. J. Quicke

**Affiliations:** 1 Department of Life Science, Imperial College London, Ascot, Berkshire, United Kingdom; 2 Department of Entomology, Natural History Museum, London, United Kingdom; American Museum of Natural History, United States of America

## Abstract

Much has been written about latitudinal trends in parasitoid diversity and biology, though it is widely recognised that they are a comparatively poorly known group. Here I show that for both braconid and ichneumonid wasps there are highly significant relationships between body size and the mean recorded latitude of species. Numbers of species per genus (surrogates of clades) peaks in the temperate zone for both families contrasting with data from the virtually complete inventories for mammals, birds and monocot plants, suggesting massive under-description of tropical parasitoid faunas. If the ichneumonoids may be expected to show similar trends to mammals, birds and other groups, the implication is that taxonomic work both in terms of active generic revisions, but also likely, the collecting and processing of museum specimens, and selection of taxa for revision, is woefully inadequate to allow latitudinal patterns in biology to be analysed.

## Introduction

One of the best known and most studied patterns in ecology is the general increase in numbers of species towards the tropics [1]. Its generality has recently been assessed in a meta-analysis of more than 600 studies [2] and was found to be upheld in nearly all cases, though with a small number of apparent exceptions [2], [3] including shore birds, penguins, freshwater zooplankton and the enormously species rich parasitic wasp family Ichneumonidae (Insecta: Hymenoptera). Such cases appear so exceptional that considerable attention has been given to them in terms of trying to explain why they behave so unusually. Here I concentrate on the last of these which has been widely cited and become textbook case.

That the enormous family Ichneumonidae was apparently less diverse at low latitudes dates back to the 1970's when Owen & Owen [4] reported results of analysing Malaise trap samples from gardens in Leicester, UK, and Freetown, Sierra Leone. This very unusual pattern soon became known as *anomalous diversity* [5], and has been replicated by a number of other studies, for example [6], [7]. So unexpected was this apparent pattern that it spurred much further work, and many hypotheses were developed to explain it, some of which have been put to various levels of testing [8], [9].

More recently, extensive collecting in a few tropical localities or surveys along transects over a wide latitudinal range, carried out in collaborations with expert taxonomists have started to indicate that actually there may not be fewer ichneumonids at lower latitudes [10]–[13] though the species abundance distributions of ichneumonids in the tropics may have exceeding long tails of rare species. Much of the associated taxonomic work, however, has been heavily focused on the most tractable taxa, such as members of the subfamilies Ophioninae and Pimplinae, which tend to be relatively large bodied, easily recognised, and potentially, not the most speciose.

Given that it now seems probable that ichneumonid wasps do not display anomalous diversity, and that the original results suggesting anomalous diversity might instead be regarded as an artefact of insufficient sampling, then any other latitudinal gradients in these parasitoid wasps that might be biased by sampling intensity, require greater scrutiny.

One of the possible explanations of apparent anomalous diversity that has been most widely cited is the *‘nasty host hypothesis’* [14], [15]. This has became widely popularised and, indeed, appears to be supported by a considerable body of evidence, some albeit rather anecdotal. The nasty host hypothesis was based in part on the general observation that tropical plants often possess higher loads of protective secondary compounds, which in turn may lead to greater host specialisation by insect herbivores. Because many of these (often larval) herbivores sequester rather than detoxify the secondary compounds, they too can gain protection from predators and parasitoids, and so specialisation of parasitoids may be higher in the tropics. This in turn can lead to rarity issues, with some of the rarer hosts effectively entering an enemy-free space.

A corollary of the nasty host hypothesis is that while parasitoids feeding on hosts that sequester potentially harmful compounds might be expected to be less common in the tropics, those attacking hosts with little or no opportunity to sequester noxious secondary plant compounds, might not be affected at all, and therefore be relatively no less abundant than other non-parasitic taxa (see [8]). This category of hosts may be exemplified by those that feed endophytically, predominantly on woody plant tissues, and these in turn are attacked by parasitoids that often display the so-called idiobiont strategy, wherein hosts are attacked at a late stage of their development, not allowed to continue developing after parasitisation and are typically consumed ectoparasitically [16], [17]. An increase in the relative proportion of idiobiont parasitoids towards the tropics has been widely noted [8], [18]. However, trends like this in such incompletely known taxa as the parasitic Hymenoptera might possibly result from other sources. For example, idiobionts, in general (excluding egg parasitoids that do not occur in the Ichneumonidae), attack hosts at a later stage in their development than do koinobionts, since the host at the time of parasitisation is a limiting resource. This might therefore mean, all other things being equal, that idiobionts might be larger bodied than koinobionts. Since small bodied taxa tend to be less well known taxonomically than larger bodied ones, samples of taxa (such as those sampled through the process of taxonomic description) from less well known regions are potentially likely to be biased towards larger-bodied, and hence idiobiont, taxa.

Santos & Quicke [19] presented species density distribution maps for both the Ichneumonidae, and for its sister family, the Braconidae, based on data in the catalogue of Yu et al. [17]. This showed the highest densities in European countries, particularly low values for some sub-Saharan African countries, and otherwise a patchwork of densities across much of the rest of the world. They also showed that there were highly significant negative correlations between absolute latitude and body size, though inclusion of subfamily as a factor showed that whereas the trend was upheld within many of the individual ichneumonid subfamilies, in the Braconidae, the overall relationship could be explained by differential representation of different subfamilies at different latitudes.

Here I explore latitudinal trends in the species richness of genera, body size and biology of the described members of the Ichneumonidae and of its sister family, the Braconidae, with approximately 24000 and 17000 described species respectively [20], [21], [22]. For both families there are significant increases in the proportions of idiobiont ectoparasitoid species towards the equator as predicted by, for example, the nasty host hypothesis. However, there are also significant increases in the proportion of idiobionts with increasing body size for both families. Given that larger bodied species are generally likely to be described before smaller bodied ones [23], this raises the possibility that the apparent latitudinal trends in biology are artefacts of biased taxonomic description. Comparisons with equivalent distributions of the far more completely known mammals and birds, and global diversity maps also for monocot plants, suggest that EITHER there is a far greater degree of anomalous diversity than perhaps appreciated, OR more likely, under-description of small-bodied tropical taxa is so extreme that it is currently impossible to be confident about any latitudinal trends in this important group of insects.

## Results

### Species richness of genera

Currently accepted genera are taken here as surrogates for clades, and therefore any latitudinal trends in the species richness of genera may plausibly be interpreted as indications of relative speciation and extinction rates and clade ages at different latitudes.

#### Braconidae and Ichneumonidae

**Figure 1:**
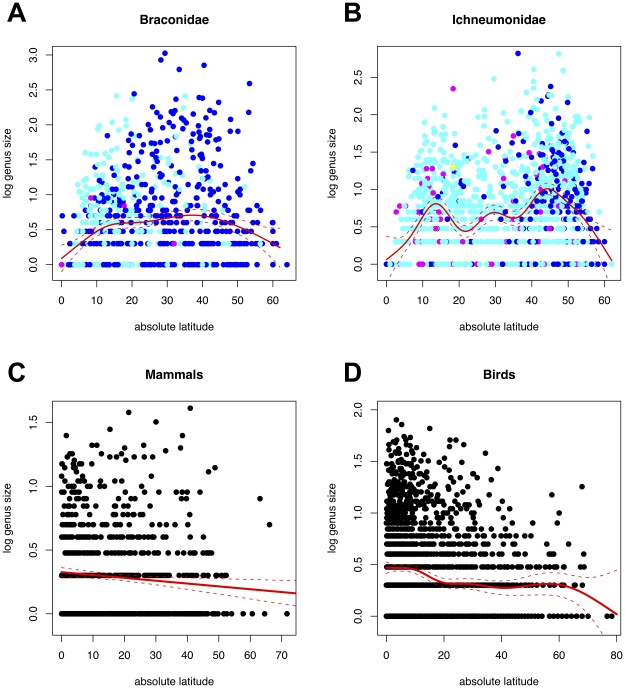
Relationships between numbers of species per genus and median absolute latitude of genera. Plots of log_10_ numbers of species per genus for all genera of: (A) Hymenoptera, Braconidae; (B) Hymenoptera, Ichneumonidae; (C) mammals; (D) birds. All plots show as a red solid line a GAM fit to the data, +/− (red dashed lines) standard errors. Figures 1 and 2 have genus data points colour coded for wasp body size (dark blue <6.5 mm, cyan = 6.6–19.5 mm, magenta = 19.6–33 mm, yellow = >33.1 mm).

Plotting the numbers of described species for each genus of Braconidae against absolute latitude (Figure 1A) shows a strongly peaked distribution with the highest species richness occurring around 40 degrees latitude, and tailing to far lower numbers towards the equator (note log base 10 scale). The fitted GAM additionally shows a small hump around 15 degrees (deviance explained 5.78% on estimated 5.034 degrees of freedom, p<2e-16). For the Ichneumonidae (Figure 1B) the low equatorial genus size effect is similarly very apparent, and the GAM fit clearly shows peaks at around 10 and 45 degrees with a smaller hump between them (deviance explained 12.2% on estimated 8.6 degrees of freedom, p<2e-16). The standardised errors (broken lines) in the middle of the distribution do not overlap with those at the peaks indicating a significant departure from unimodality.

The genera of Braconidae and Ichneumonidae in Figures 1A, B are colour-coded according to mean adult body length, and in both cases, the mean sizes of the described species of wasps in genera that are distributed at low latitudes are conspicuously larger than those at higher latitudes. The same size thresholds were used for both families and a clear difference is apparent between families with the described Ichneumonidae being markedly larger on average than the Braconidae.

#### Comparisons with birds and mammals

The species richnesses of genera of birds and mammals versus latitude are shown in Figures 1C–D, and both show maximal values very close to the equator, with GAM fits maximal at the equator. For birds the GAM is somewhat humped, though with no statistical confidence, at high latitudes (apparently partially due to sea birds - data not shown) (deviance explained 3.24% on estimated 5.82 degrees of freedom, p<2e-16). For mammals, the GAM fit is effectively straight showing that the data can be adequately modelled with a linear model (deviance explained 0.74% on estimated 1 degrees of freedom, p<2e-16).

### Relationships between biology and latitude and body size

#### Braconidae and Ichneumonidae

**Figure 2:**
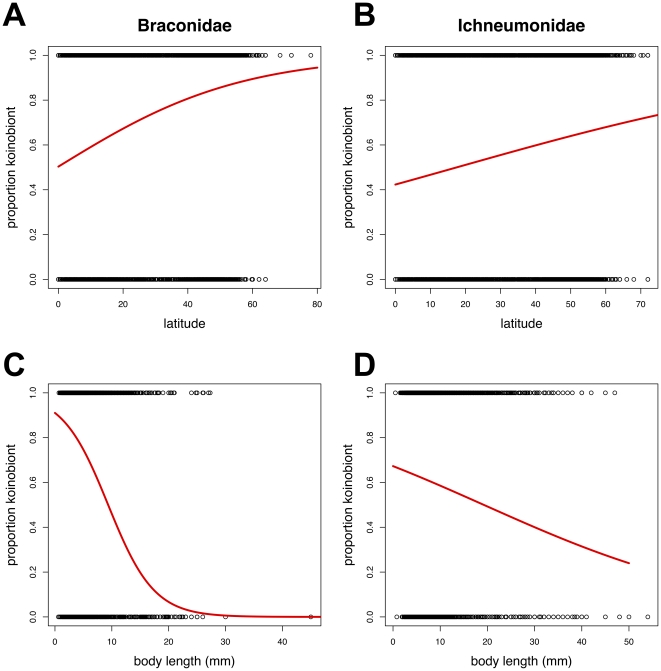
Relationship between biology, latitude and body size. Logistic regression plots of proportions of koinobiont species versus absolute latitude for braconid and ichneumonid (A, B) wasps, and proportions of koinobiont species and maximum body length (C, D).

The relationship between biology (idiobiont versus koinobiont) and both latitude and body length was examined using logistic regression. There was no significant interaction term and the minimum adequate model had only latitude and body size as separate significant explanatory variables. For both families, the proportion of described koinobionts significantly increases with latitude as shown in Figures 2A and 2B (Braconidae: residual deviance 16396 on 14952 degrees of freedom, p<2e-16; Ichneumonidae: residual deviance 24710 on 18473 degrees of freedom, p<2e-16). Biology is also significantly related to body size for both families (Figures 2C, D) with the proportion of koinobionts decreasing with increased body length latitude (Braconidae: residual deviance 15167 on 14952 degrees of freedom, p<2e-16; Ichneumonidae: residual deviance 24943 on 18473 degrees of freedom, p<2e-16).

### Mapping genus richness by political region

The numbers of genera recorded from each country of the world provides a summary of the overall geographic spread of a group's diversity while being less sensitive to the level of recording of each species, and therefore seems more appropriate to relatively undescribed taxa such as the ichneumonoid wasps.

#### Braconidae and Ichneumonidae

**Figure 3:**
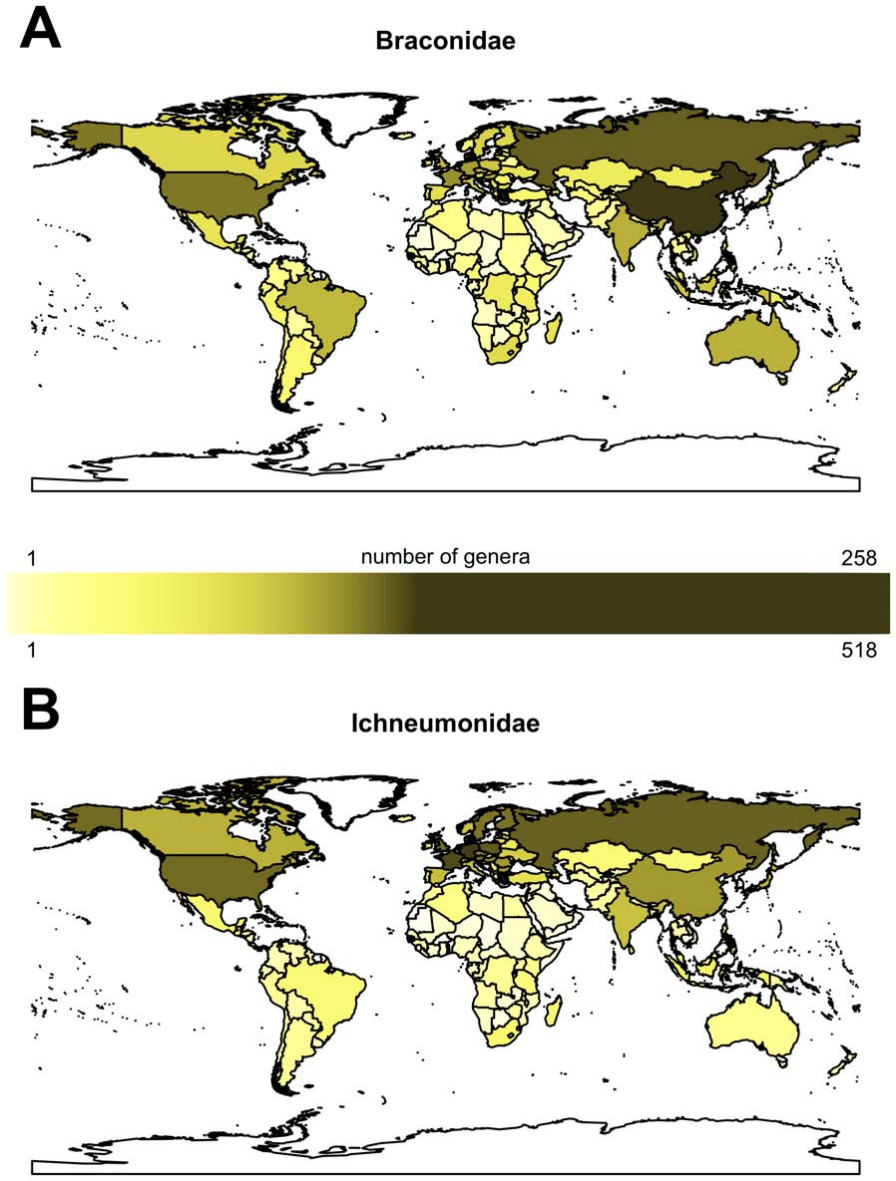
World maps shaded to show numbers of wasp genera recorded per country. Braconid (A) and ichneumonid (B) wasps.

Shaded maps for the Braconidae and Ichneumonidae are presented in Figures 3A and 3B respectively. For both families the highest generic numbers are recorded for countries in European, especially the UK and some central European ones, and in the case of the Braconidae, also China.

#### Comparisons with mammals, birds and monocot plants

**Figure 4:**
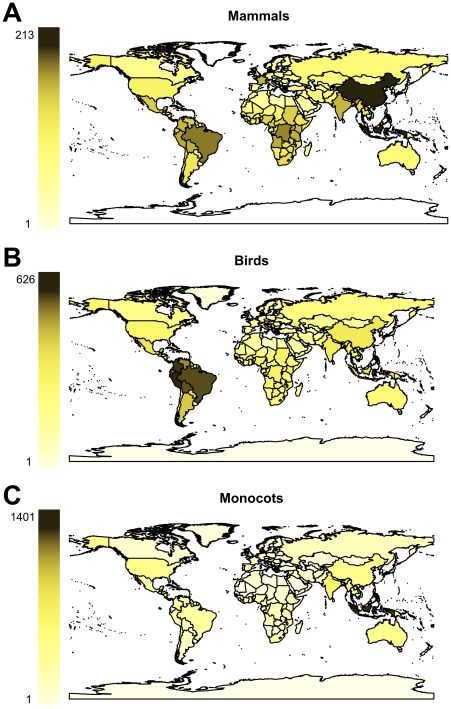
World maps shaded to show numbers of mammal, bird and monocot genera recorded per country.

Equivalent colour coded maps for three very well studied groups of organisms, mammals, birds (breeding ranges) and monocot plants (excluding Poaceae) are shown in Figures 4A–4C respectively. Each major group has a distinctive pattern of genus-level richness with mammal genus richness dominated by China and South East Asia, birds in tropical South American countries and monocots in South East Asia. All three groups, however, are generally more generically rich in tropical regions of South America, Africa and Asia.

### Adding estimation of undescribed species to latitudinal patterns

To try to compensate for this systematically biased under-description, I inflated the numbers of species per genus according to the significant explanatory variables from the analysis of the Jones et al. [24] based on the changes that occur in the number of recognized species in genera of braconids following taxonomic revision. Jones et al. showed that for the Braconidae there was a relationship between the relative change in the number of species of a genus after a taxonomic revision, insect body size and geographic region; this change following revision was referred to as the ‘multiplier’. The data of Jones et al. were reanalysed using mean absolute latitude of species of each genus instead of geographic region. The resulting minimal linear model (there was no significant interaction term) included 2 significant slopes and a significant intercept: −0.03 * absolute latitude (degrees)+0.70 log_10_(body length in mm)+4.4.

**Figure 5:**
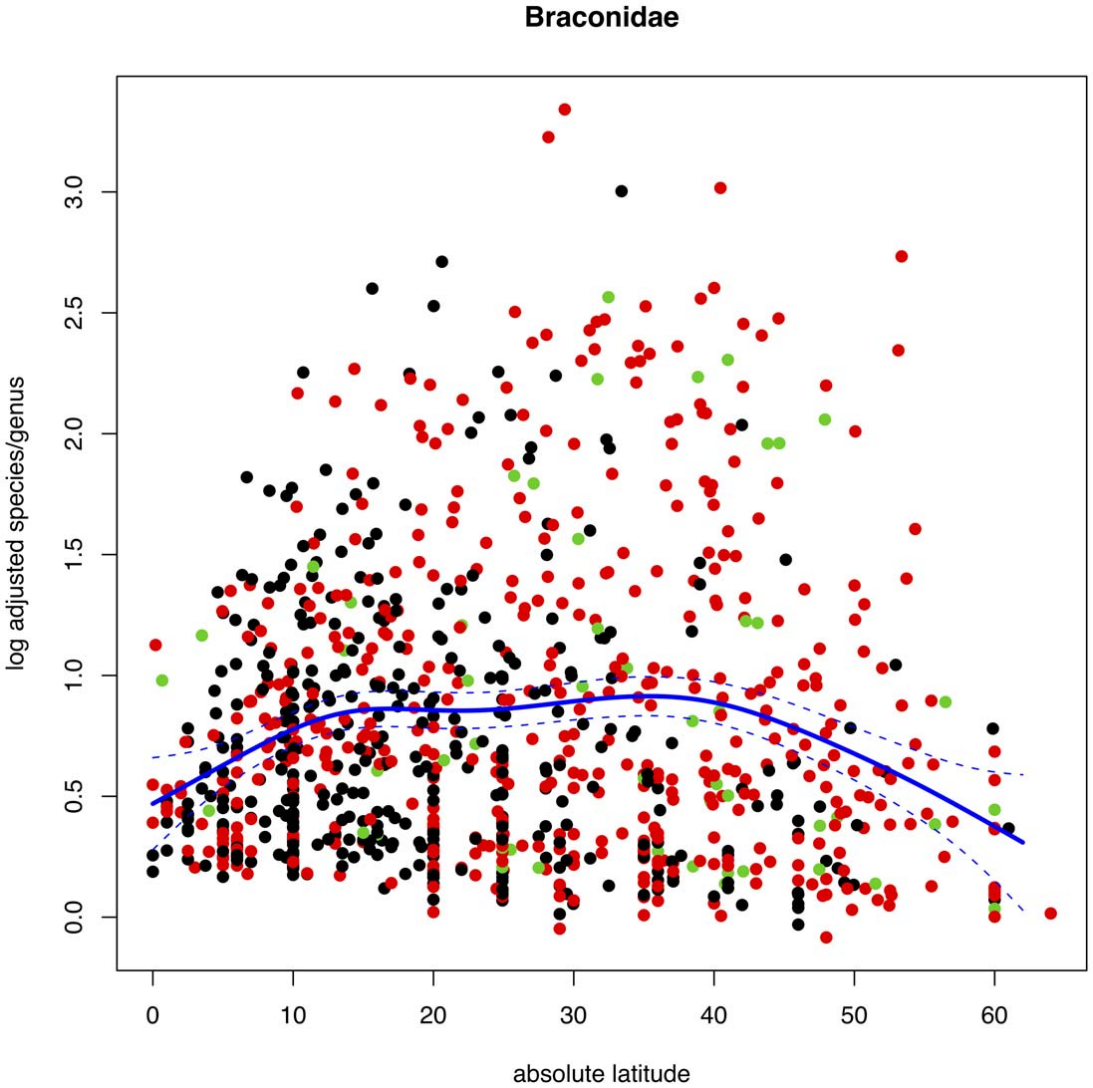
Relationship between median absolute latitude of genera and adjusted numbers of species per genus. The numbers of species per genus for all genera of Braconidae are transformed according the relationship found between the change in numbers of recognized species following taxonomic revision and body size and latitude (based on data in Jones *et al.* 2009). Genera are colour-coded according to biology with idiobionts black, koinobionts red and the Euphorinae excluding the koinobiont Meteorini green as the biology of these is intermediate between idiobionts and koinobionts in several respects.

This model was then applied as a transformation to the genus species richness versus latitude data shown in Figure 1A for the Braconidae; no comparable study of the relative change in ichneumonid genera following revision is available. The transformed plot is shown in Figure 5, which additionally indicates biology. Whilst the estimated magnitudes of genus size are increased markedly (note the log scale), there is no conspicuous change in the shape of the distribution, which remains essentially unimodal with a peak between 30 and 40 degrees.

Jones et al. [24] also examined the implications of the numbers of species represented in revisionary studies by just single or precisely two individuals. With these two values and the total number of species following revision, they estimated for each taxon, the number of undescribed species using the Chao-1 non-parametric estimator [25], [26]. Re-analysis of the Chao-1 estimates relative to the known accepted number of valid species following taxonomic revision as a function of log(body size), absolute latitude and their interaction gave the same result as in Jones et al., in that the only significant term was the intercept. Thus, whilst using this to transform the data further would increase the overall estimates of genus size versus latitude, it would not change the shape of the distribution.

## Discussion

Potential latitudinal gradients in body size between species of insects have received relatively little attention despite the probably misleading impression that most of the largest species exist in tropical locations - there are, afterall, many more species of most insect groups within the tropics. Cushman et al. [27] noted that within a strictly European context, ant assemblages at higher latitudes included fewer small bodied species and proposed several possible mechanisms for this observation. In terms of interspecific studies this was followed by Hawkins [28] who found no significant trend for bees of North America, and Hawkins & Lawton [29] who examined latitudinal gradients in body size of butterflies on a global basis, and concluded that when trends were present, these were mostly accountable for by the differential representation of butterfly families of different mean size at different latitudes. This result is essentially the same as what Santos & Quicke [19] showed for both ichneumonids and braconids, with highly significant negative relationships between body size and absolute latitude for both families. However, for the Braconidae, taking subfamily into account completely accounted for the significance, i.e. under-representation of subfamilies characterised by small body size among the tropical taxa accounted for the effect. Whether this is due to an artefact of the small tropical species having been grossly under-described, or real differences in representation with latitude or a combination of the two is not instantly apparent. Interestingly, in the ichneumonids, which generally have larger body sizes than braconids, taking subfamily into account did not eliminate the overall latitudinal trend in body size, and indeed, there were similar trends towards larger-bodied species at lower latitudes in most of those subfamilies that showed significant trends. This could be accounted for similarly by either a bias in description (likely), real trends, or a combination of the two.

Here I show that the current level of taxonomic treatment for two very speciose families of insects, show strong latitudinal patterns of species richness that differ markedly from the patterns seen in far better studied mammals and birds. Instead of peaking at or near the equator, apparent richness declines markedly at low latitudes. Consideration of body size versus latitude relationships suggests that either smaller bodied species of both Ichneumonidae and Braconidae are either under-represented in low latitude faunas, or that they have been systematically under-described. Evidence for the latter was provided by Jones et al. [24] who showed that when groups were revised, significantly more new species were added when body size was small. In general, larger bodied and more widespread species are more likely to be described earlier (see, for example, [23], [30]).

Giam et al. [31] have indicated that even for well studied groups of animals and plants, there are still significant numbers of new species awaiting description, and that these are predominantly likely to be found in the least anthropogenically disturbed moist tropical forests of the Neotropics, Afrotropics and Indomalaya. Such species are likely to have small distribution ranges. For groups such as the Ichneumonoidea where the great majority of extant species are almost certainly undescribed, understanding major latitudinal and other longitudinal trends will necessarily require, at the least, more large biodiversity studies at a range of tropical sites, with equal weight being given to taxa of all body sizes. Indeed, Veijalainen et al. [32] have shown, based on extensive Malaise trap sampling in Peru, Costa Rica and the USA, that the relative proportions of ichneumonid subfamilies is roughly similar across the range, and that contrary to previous assumptions, several koinobiont subfamilies were represented by very large numbers of individuals in the Amazonian basin samples. Given the relative dearth of morpho-taxonomic expertise and background taxonomic frameworks for many tropical taxa, such studies are likely to have to have a large molecular component such as through DNA barcoding, e.g. Smith et al. [33] and Veijalainen et al. [34].

## Materials and Methods

Data on the distributions and body sizes of ichneumonoid species were extracted from the Taxapad database [20]. Mean latitudes of countries were largely derived from http://www.maxmind.com/app/country_latlon. Body size for ichneumonoids was taken as the maximum recorded body size.

For the Ichneumonoidea data, every record of a species from a country is provided [20], and additionally for some large countries (e.g. USA and Russia) records are provided on a provincial basis (e.g. individual states of the USA). To reduce potential inflation of the latitude by having potentially multiple occurrences for these single countries, a single record assigned the mean latitude of the country was given, with the exceptions that species records from overseas dependencies or states (e.g. St Helena of the UK, Hawaii of the USA) or widely separated states (e.g. Alaska of the USA) were treated separately. For calculations at species level the mean of the latitudes of all the countries (or widely separated provinces as above) where each species has been recorded was taken as the species' latitude. For calculations at genus level, the mean latitude of all the species' mean latitudes was used.

For calculations using revised estimates of species richness based on the relationships between body size and latitude the relative change in numbers of species recognised following taxonomic revisions of genera, I used the data set and regressions from Jones et al. [24] with the most inclusive source publications filter.

The biologies, i.e. idiobiont or koinobiont, of braconid and ichneumonid species are largely phylogenetically constrained, and were classified on the basis of the known biologies of members of their subfamilies, or occasionally lower divisions if variation was known to occur. Within the Braconidae the Euphorinae sensu stricto (also taken to include Ecnomiinae and Neoneurinae: see Belshaw & Quicke [35] and Sharanowski et al. [36]) interpretation of biology in terms of idiobiosis is somewhat ambiguous and for some analyses was treated as a 3rd state.

For comparison with the Ichneumonidae and Braconidae data, I analysed three data sets of taxa whose current taxonomy is far more complete, the mammals, birds and monocotyledonous plants. The mammal data came from the publicly available PanTHERIA data base [37]; the bird data are those analysed by Orme et al. [38] and available from i.owens@imperial.ac.uk; data on monocots were downloaded as flat text files from http://apps.kew.org/wcsp, standardized, combined and parsed to extract necessary data.

All statistical modeling, parsing and calculations were carried out using the statistical computing language R [39]. Interpretation of species per genus versus latitude was assisted by use of generalised additive models (GAMs) using the *mgcv* library in R.
